# Mesenchymal stem cells: A new therapeutic tool for chronic kidney disease

**DOI:** 10.3389/fcell.2022.910592

**Published:** 2022-10-04

**Authors:** Jiali Wang, Yongda Lin, Xiutian Chen, Yiping Liu, Tianbiao Zhou

**Affiliations:** Department of Nephrology, the Second Affiliated Hospital, Shantou University Medical College, Shantou, China

**Keywords:** mesenchymal stem cells, mesenchymal stem cell-derived extracellular vesicles, chronic kidney disease, diabetic nephropathy, lupus nephritis, autosomal dominant polycystic kidney disease

## Abstract

Chronic kidney disease (CKD) has a major impact on public health, which could progress to end-stage kidney disease (ESRD) and consume many medical resources. Currently, the treatment for CKD has many flaws, so more effective treatment tools are urgently required for CKD. Mesenchymal stem cells (MSCs) are primitive cells with self-renewal and proliferation capacity and differentiation potential. Extensive preclinical and clinical data has shown that cell-based therapies using MSCs can modulate immunity, inhibit inflammatory factors, and improve renal function in CKD, suggesting that MSCs have the potential to be a new, effective therapeutic tool for CKD. In this review, we will describe different kinds of MSCs and MSCs products for the treatment of CKD in experimental models and clinical trials, potential signaling pathways, therapeutic efficacy, and critical issues that need to be addressed before therapeutic application in humans.

## Introduction

Chronic kidney disease (CKD) refers to chronic kidney structure and function obstacles for three or more months for any causes. CKD may lead to many complications, such as hyperkalemia (Gennari and Segal, 2002), metabolic acidosis (Uribarri et al., 1995), hypertension (Stefanski et al., 1996), anemia (Eschbach, 1991), and sexual dysfunction (Procci et al., 1981). Complications of CKD have a major impact on the quality of life of patients and can increase the risk of mortality (Webster et al., 2017). According to Global Burden Assessment Report, the number of people dying of CKD has increased in recent years (Xie et al., 2018; Wong, 2021). The prevalence of CKD is rising as the incidences of the main risk factors for CKD, including DM and hypertension, are sharply increasing, leading to a rising prevalence of CKD. Therefore, CKD has been putting pressure on medical resources as a critical cause of global mortality (Ene-Iordache et al., 2016; Simeoni et al., 2021).

Common causes of CKD include poorly controlled diabetes mellitus (DM), hypertension, glomerular disease, glomerulonephritis, and autosomal dominant polycystic kidney disease (Webster et al., 2017). Statins, antiplatelet agents, renin-angiotensin system inhibitors, sodium-glucose cotransporter 2 (SGLT2) inhibitors, lifestyle changes, and other treatments are used to slow the progression of CKD (Heerspink et al., 2018; Simeoni et al., 2021). However, new therapies are limited. When CKD progress cannot be prevented, it will evolve into end-stage renal disease (ESRD). Those with ESRD, compared with patients with CKD, had a significantly higher frequency of vascular disease, cognitive impairment, and depression (Murali et al., 2020). The current treatment approaches for ESRD are dialysis and kidney transplantation, which consume a lot of health care resources (Webster et al., 2017). Moreover, dialysis may lead to deterioration of the quality of life and kidney transplantation may carry the risk of severe complications (Ene-Iordache et al., 2016; Webster et al., 2017). Therefore, novel therapies for CKD are required.

A hallmark of CKD is renal fibrosis. Renal fibrosis includes glomerulosclerosis and tubulointerstitial fibrosis, which can be divided into four phases, including cellular injury, fibrogenic signaling activation, fibrogenic execution, and destruction (Genovese et al., 2014). Endothelial damage and dysfunction, mesangial cell proliferation, podocyte destruction, and tubular atrophy are essential contributors to CKD progression (Webster et al., 2017). During the CKD progression, epithelial-mesenchymal transition (EMT) is a crucial process. The key inducer of EMT is transforming growth factor-β1 (TGF-β1) (Wu et al., 2014) and the important symbols of EMT are E-cadherin loss and overexpression of α-smooth muscle actin (α-SMA). Extracellular matrix (ECM) deposition, including over-expression of fibronectin, collagen I, and collagen IV is an important feature of kidney fibrosis. In addition, macrophage aggregation, myofibroblast activation, and loss of peritubular capillary are imperative factors in CKD progression (Webster et al., 2017). Some signaling pathways, such as TGF-β, Mitogen-activated protein kinase (MAPK), Wnt/β-catenin, PI3K/Akt, JAK/STAT, and Notch pathways, are closely related to CKD (Edeling et al., 2016; Liu et al., 2017; Hu et al., 2018). Therefore, regenerating specific renal cells and blocking the pathway of CKD progression is vital for CKD treatment.

In many previous studies, mesenchymal stem cells (MSCs) have been found to be safe and feasible for treating cardiomyopathy (Chin et al., 2010; Chin et al., 2011; Chin et al., 2021), multiple myeloma (Teoh et al., 2016), and DM (Li et al., 2021). It is also reported as an effective treatment for fibrosis diseases, like liver fibrosis (Eom et al., 2015), lung fibrosis (Barczyk et al., 2015), as well as myocardial fibrosis (Jin et al., 2020). MSCs are attractive candidates for kidney repair therapy because they can promote recovery and prevent kidney failure (Wong et al., 2014).

This review includes the role of different kinds of MSCs and MSCs products such as d extracellular vesicles from MSCs (MSCs-EVs) for treating CKD in preclinical and clinical research and possible mechanisms, along with prospects and challenges in clinical application.

### Characteristics of different kinds of MSCs

MSCs are plastic-adherent cells and can be expanded to yield many cell numbers. However, there is no specific marker found to characterize MSCs better so far. The International Society of Cell Therapy (ISCT) has proposed the minimal criteria for MSCs definition, which includes: adherence to plastic, tri-lineage, and differentiation potential; positive for CD105, CD73, and CD90 marker expression while being negative for CD45, CD34, CD14 or CD11b, CD79a or CD19, and HLA-DR expression on the cell surface; the ability to differentiate into osteoblasts, adipocytes, and chondroblasts *in vitro* culture conditions (Dominici et al., 2006). MSCs include bone marrow mesenchymal stem cells (BMMSCs), adipose-derived MSCs (ADMSCs), umbilical cord blood mesenchymal stem cells (UCMSCs), human placenta-derived MSCs (PDMSCs), Wharton’s jelly-derived MSCs (WJMSCs), dental mesenchymal stem cells (DMSCs), and so on (Pittenger et al., 1999).

The most commonly used cell types for CKD are BMMSCs. BMMSCs have low expression of MHC I and MHC II, and thus can avoid attack by allogeneic T cells. Studies have shown that BMSCs can differentiate into glomerular mesangial cells. However, BMMSCs are difficult to purify efficiently and quickly. As a result, researchers examined different types of MSCs for kidney regeneration.

Among the various sources, ADMSCs derived from adipose tissue have become promising candidates. They have characterized surface markers including CD44/CD90 or CD34/kinase insert domain receptor (Zhu et al., 2013). ADMSCs, like BMMSCs, may differentiate into distinct lineages and have the potential to be used therapeutically to restore tissues that have been injured. In addition, like BMMSCs, ADMSCs also have minimal immunogenicity and operate as immunomodulators (Puissant et al., 2005). ADMSCs exhibit more stronger immunomodulatory effects than BMMSCs due to variations in cytokine production. ADMSCs had a greater degree of cytokine release, particularly interleukin-6 (IL-6) and TGF-β1, which have been linked to the immunomodulatory actions of MSCs. This is due to ADMSCs having increased metabolic activity when compared to BMMSCs. In addition, ADMSCs are more effective than BMMSCs at suppressing the proliferation of peripheral blood mononuclear cells and preventing the development of dendritic cells (Melief et al., 2013). Besides, ADMSCs have other advantages, including widely available sources, easy access, minimal injury, rapid proliferation, easy purification, and a lack of ethical concerns (Katsuno et al., 2013). Adipose tissue is an excellent source of MSCs, but ADMSCs are not as effective at restoring kidney function as BMMSCs (Ashour et al., 2016).

UCMSCs also provide an alternative source of MSCs. UCMSCs could transform medical waste into a valuable product with clinical applications (Musiał-Wysocka et al., 2019). Immune rejection in allogeneic transplants is unlikely because UCMSCs are less immunogenic than MSCs from other sources (Li et al., 2014). In addition, UCMSCs proliferate more rapidly than BMMSCs or ADMSCs (Fazzina et al., 2016). Moreover, UCMSCs show no signs of senescence despite multiple passages, suggesting they can be manufactured in large numbers without losing potency (La Rocca et al., 2009).

In contrast to ADMSCs and BMMSCs, where MSCs are affected by the donor’s age, the placenta is an excellent source for cell therapy (Shigeno and Ashton, 1995). Additionally, PDMSCs can be collected, cultured, and evaluated after childbirth and the placenta removed from the uterus. PDMSCs could offer numerous sources, have low immunogenicity, and have no ethical concerns, making them a promising cell therapy source. Therefore, the proliferation potential of PDMSCs is greater than that of BMMSCs (Liu et al., 2019).

In conclusion, BMMSCs are the cell types that are most frequently used for CKD, however, BMMSCs are challenging to purify quickly and effectively. ADMSCs, UCMSCs, and PDMSCs have consequently emerged as promising options. ADMSCs have several advantages over BMMSCs, such as easy purification, quick proliferation, high cell viability, and convenient supply and more potent immunomodulatory effects (Katsuno et al., 2013; Melief et al., 2013). Compared to BMMSCs or ADMSCs, UCMSCs multiply more quickly, and they can be produced in enormous quantities without losing their effectiveness (La Rocca et al., 2009). PDMSCs have a wide range of sources, minimal immunogenicity, and no ethical issues. Additionally, PDMSCs have a higher proliferative potential than BMMSCs (Liu et al., 2019).

## MSCs and MSCs-EVs with CKD in preclinic study

### MSCs and CKD in preclinic study

Animal models have been widely used to better understand the pathogenesis and underlying mechanisms of CKD. Mice and rats are the most commonly used models for studying CKD and potential therapeutic targets. Typical animal models for glomerular disease include the COL4A3 gene knockout model, 5/6 NPX (nephrectomy) model, diabetic mellitus model, and Lupus nephritis (LN) model. Common models for tubule-interstitial fibrosis include unilateral ureteral obstruction (UUO) model, albumin-overloaded model and polycystic kidney disease (PKD) model. We reviewed the studies in those fields. Also, the safety and efficacy of MSCs in various CKD models are discussed in this section.

One of the most commonly used experimental models of CKD is the remnant kidney model. In rats, 5/6 NPX is typically used to mimic human CKD. Villanueva S et al. transplanted ADMSCs to 5/6 NPX rats and found that the group treated with ADMSCs had a higher level of protecting factors, such as epitheliogenic, bone morphogenetic protein 7 (BMP-7), and vascular endothelial-derived growth factor (VEGF). Also, the levels of damage markers in renal fibrosis, including ED-1 and α-SMA are decreased. Additionally, the study showed that Oct-4, a marker of renal ADMSCs, increased after ADMSCs therapy (Villanueva et al., 2013).

MSCs also exert protective effects in diabetic mellitus models in mice. In order to test the effectiveness of a single dose of BMMSCs in the experimental model of type 1 diabetic mice, Ezquer et al. injected 0.5×10^6^ BMMSCs into Streptozotocin (STZ) induced type 1 diabetes C57BL/6 mice. The kidneys treated by BMMSCs showed a more normal appearance. Besides, BMMSCs were found to revert hyperglycemia, glycosuria, and microalbumin (Ezquer et al., 2008). By contrast, Lee et al. used a larger number of BMMSCs in a type 2 diabetes model. It was observed that human BMMSCs prevented further blood glucose increases but did not revert hyperglycemia. One possible implication of this is that MSCs exert therapeutic effects in a type 1 diabetic animal model but have preventive effects in a type 2 diabetic animal model induced by STZ. The study also showed that human BMMSCs decrease mesangial thickening and macrophage infiltration, which are important stages in CKD. Single cells were found both in pancreatic islets and glomeruli, indicating that BMMSCs are selective homing (Lee et al., 2006). In another study, STZ-induced DN rats were infused with 2×10^6^ human UCMSCs *via* the tail vein at week 6. The study shows that UCMSCs increase the level of growth factors, fibroblast growth factor (FGFs), HGF, and VEGF but decrease levels of IL-6, interleukin-1β (IL-1β), tumor necrosis factor alpha (TNF-α) and TGF-β, thus improving the renal function (Xiang et al., 2020).

Murine models are used in the majority of LN research. MRL and CD95 mutants are widely used to develop proteinuria, lymphoproliferation, and other features similar to human LN (Mcgaha and Madaio, 2014). The inflammatory microenvironment of SLE patients increases NF-κB expression in glomerular endothelial and mesangial cells, while NF-κB overexpression can induce the expression of inflammatory cytokines, chemokines, adhesion molecules, and inflammatory enzymes, creating a vicious cycle (Liu et al., 2019). NF-κB signaling pathway activation could be downregulated in MRL/lpr mice after infusion of human PDMSCs by reducing the level of NF-κB mRNA and phospho-NF-κB p65. Also, TNF-α, plasminogen activator inhibitor-1 (PAI-1), and ICAM-1 expression in MRL/lpr mouse kidneys was observed to decrease (Liu et al., 2019). In a study by Ruan et al., human UCMSCs were given *via* intravenous injections for 4 weeks to B6. Fas mice. The results showed decreased levels of antinuclear, anti-histone, and anti-dsDNA antibodies within the MSCs treatment group. In the MSCs group, the 24-h urine protein level was lower than in the lupus group, indicating that renal function improved. Compared to the lupus group, renal pathology was ameliorated by UCMSCs treatment, such as glomerular mesangial cell proliferation, tubulointerstitial fibrosis reduction, and diminished immune complex deposition in the glomeruli (Ruan et al., 2014).

The COL4A3 gene knockout mouse is a model for analyzing the pathogenesis of Alport syndrome. In a model of COL4A3-deficient mice, BMMSCs injections to COL4A3-deficient mice can prevent peritubular capillary loss through the production of VEGF mRNA. Hepatocyte growth factor (HGF) and tumor necrosis factor stimulated gene-6 (TSG-6), as paracrine factors of BMMSCs, may contribute to this positive result, while BMMSCs differentiation is unimportant in this process (Ninichuk et al., 2006).

The UUO model is used to mimic tubulointerstitial fibrosis. Obstruction of the urinary tract causes hydronephrosis, interstitial inflammatory infiltration, and tubular cell death caused by apoptosis and necrosis (Docherty et al., 2006). Many studies have proved the effectiveness of the BMMSCs by the UUO model. In a UUO model in mice, cultured BMMSCs transplanted through the tail vein can attenuate cell death, macrophage infiltration, and renal fibrosis. In addition, Ki67, a marker used to evaluate cell proliferation was increasing while CD68-positive macrophage infiltration was decreasing (Xing et al., 2019). In another UUO model, the UUO + BMMSCs + melatonin (MT) group had the lower gene and protein expressions of TGF-β1, α-SMA, and TNF-α but higher expression of E-cadherin (Saberi et al., 2019). In conclusion, BMMSCs infusion can increase cell proliferation, and decrease peritubular capillary loss and macrophage infiltration in the UUO model. BMMSCs were injected into the UUO rat through the cava vein and da Silva et al. reported that both BMMSCs and their conditioned medium could decrease levels of collagen I, TNF-α, caspase 3, α-SMA, and proliferating cell nuclear antigen (PCNA) and finally decrease EMT and fibrosis area (Da Silva et al., 2015).

BMMSCs could also reduce tubular EMT and renal fibrosis in albumin-overloaded mice. A study reported that BMMSCs can decrease levels of tubular CCL-2, CCL-5, TNF-α, α-SMA, fibronectin (FN), and collagen IV and finally reduce tubular EMT and renal fibrosis in albumin-overloaded mice. Also, research has shown that BMMSCs delivered *via* the renal artery can lower BUN and UACR (Wu et al., 2014).

Genetically engineered murine models are widely used to mimic human PKD. Franchi et al. were the only researchers to use allogeneic MSCs in a PKD rat model. They demonstrated the beneficial effect of a single intravenous infusion of allogeneic MSCs on systolic blood pressure and fibrosis. Although the infusion had no effect on cyst size or number, it did improve cortical and parenchymal vasculature density, which resulted in better tubular function and creatinine clearance in the intervention group in the autosomal recessive PKD (ARPKD) model (Franchi et al., 2015). Furthermore, 4 weeks after transplantation, some of the MSCs that remained in the kidney developed endothelial or tubular characteristics (Franchi et al., 2015). While it is a genetic model of ARPKD, its features are quite similar to autosomal dominant polycystic kidney disease, the most common form of PKD affecting adults (Lager et al., 2001).

In conclusion, MSCs exert renal protective effects in 5/6 NPX rats, diabetic mellitus models in mice, MRL/lpr mice, COL4A3-deficient mice, UUO mice, albumin-overloaded mice, and PKD rat models by increasing levels of BMP-7, VEGF, FGFs, and HGF. Also, the levels of damage markers in renal fibrosis, including ED-1 α-SMA, IL-6, IL-1β, TNF-α, TGF-β, NF-κB mRNA, CCL-2, CCL-5, collagen I, FN, collagen IV, and PCNA are decreased (Ninichuk et al., 2006; Ezquer et al., 2008; Villanueva et al., 2013; Da Silva et al., 2015; Franchi et al., 2015; Liu et al., 2019; Xiang et al., 2020). Despite promising results in animal models, studies in CKD cats have not replicated the efficacy of MSCs treatment reported in experimentally induced CKD models (Quimby et al., 2016). A study in eight CKD cats suggested that allogeneic MSCs from cryopreserved adipose had no improvement after administration (Quimby et al., 2016). One possible explanation for the disparate outcomes in cats is that uremia impairs the function of allogeneic MSCs. Zhu et al. transplanted ADMSCs into pigs with renal artery stenosis and found that ADMSCs could raise the levels of growth factors HGF, VEGF, and their receptors, Flk-1, and Flt-1, in the pigs’ bodies. The transmural cortical microvascular density is also increasing in the therapy group. These data confirm that ADMSCs can attenuate kidney injury in the setting of renal artery stenosis (Table 1) (Zhu et al., 2013).

**TABLE 1:** Summary of preclinical data of MSCs in CKD.

Author, year	Stem cell type	Animal model	Groups	Handling methods	Treatment effect
Villanueva et al. (2013)	ADMSCs	5/6 NPX rats	1 Sham group	0.5 × 10^6^ ADMSCs or MSCs CM alone	↓plasma creatinine, damage markers ED-1 and α-SMA
			2 NPX group		
			3 NPX rats injected with 450 μL of X-VIVO™ medium (without MSCs)		↑Pax-2, BMP-7, and VEGF, Oct-4
			4 NPX rats injected in the tail vein with 450 μL (0.5 × 10^6^ cells) of ADMSCs in X-VIVO™ medium		
Ezquer et al. (2008)	BMMSCs	STZ-induced type 1 diabetes C57BL/6 mice	1 Sham group	0.5×10^6^ MSCs *via* the tail vein	↓ blood glucose levels
					↑normal beta-pancreatic islets
			2 MSCs group		Reversion of hyperglycemia and glycosuria remained for 2 months at least
					More normal glomeruli appearance
					Reverts microalbuminuria and precludes renal structural damage
Lee et al. (2006)	BMMSCs	STZ-induced diabetic NOD/scid mice	1 control mice (Normal)	2.5 × 10^6^ MSCs *via* left cardiac ventricle	↓mesangial thickening and macrophage infiltration
			2 STZ		BMMSCs prevented further blood glucose increases but did not revert hyperglycemia
			3 STZ + hMSCs		Single cells were found both in pancreatic islets and glomeruli
Xiang et al. (2020)	UCMSCs	STZ-induced DN rats	1 LG group: Dglucose 5.5 mmol/L	2 × 10^6^ MSCs *via* the tail vein	↓proteinuria, Scr, BUN, IL-6, IL-1β, TNF-α and TGF-β
			2 HG group: d-glucose 30 mmol/L		
			3 HG + 25% UCMSCs-CM group		
			4 HG + 50% UCMSCs-CM group		
			5 HG + 100% UCMSCs-CM group		↑Ccr, FGFs, HGF, and VEGF
			6 HG + 25 μg/ml UMSCs-Exo group		
			7 HG + 50 μg/ml UCMSCs-Exo group		
			8 HG + 100 μg/ml UCMSCs-Exo group		
Liu et al. (2019)	PDMSCs	a model of LN: MRL/lpr mice	1 the control group, age-matched BALB/C mice	1 × 10^6^ MSCs/300 μL of saline IV	↓NF-κB mRNA, phospho-NF-κB p65, TNF-α, PAI-1, and ICAM-1 expression
			2 the vehicle group, untreated MRL/lpr mice		
			3 the LEF group		
			4 the MSCs group		
Ruan et al. (2014)	UCMSCs	B6.Fas mice	1 normal control (C57BL/6 mice) group	Mice in the B6.Fas mouse high-dose group were injected with 2 × 10^6^ labeled cells, mice in the B6.Fas mouse medium-dose group were injected with 1 × 10^6^ labeled cells, and mice in the B6.Fas mouse low-dose group were injected with 0.5 × 10^6^ labeled cells	↓antinuclear, anti-histone, anti-dsDNA antibodies, tubulointerstitial fibrosis and immune complex deposition in the glomeruli
			2 model (B6.Fas mice) group		
			3 low-treatment (B6.Fas mice) groups		↑glomerular mesangial cell proliferation
			4 medium-treatment (B6.Fas mice) groups		
			5 high-dose treatment (B6.Fas mice) groups		
Ninichuk et al. (2006)	BMMSCs	COL4A3-deficient mouse (Alport disease model)	1 Wild type group	MSCs (1 × 10^6^) or vehicle *via* tail vein	↓BUN, Scr, glomerulosclerosis and renal fibrosis
			2 Collagen4A3 −/− + saline group		→EVGF, BMP
			3 Collagen4A3−/− + MSCs group		
Xing et al. (2019)	BMMSCs	UUO mice	1 MSCs group	500 μL MSCs (10^6^ cells/mL) *via* the tail vein	↓CD68-positive macrophage, PTC loss, renal tubulointerstitial injury and fibrosis
			2 DMEM group		↑Ki67, α-SMA
			3 UUO group		
Saberi et al. (2019)	BMMSCs	UUO rats	1 Sham group UUO group	1 × 10^6^/1 ml PBS MSCs *via* lateral tail vein	↑E-cadherin
			2 UUO + BMMSCs group		↓TGF-β1, α-SMA and TNF-α
			3 UUO + BMMSCs + MT group		MT enhance homing effect and survival of transplanted BMMSCs
Saberi et al. (2019)	BMMSCs BMMSCs-CM	UUO rats	1 Sham group	1 ×10^6^ MSCs or MSCs-CM (500 μL) *via* the cava vein	↓Col1a I, TNF-a, caspase 3, PCNA, a-SMA, EMT and fibrosis area
			2 UUO group		
			3 UUO + MSCs group		
			4 UUO + CM group		
Wu et al. (2014)	BMMSCs	albumin-overloaded mice	1 UNX group	1×10^6^ cells/mouse	↓BUN, UACR, collagen IV andα-SMA message RNAs, tubular CCL-2, CCL-5, TNF-a over expression, a-SMA, FN and collagen IV, tubular EMT
			2 UNX + MSCs group		
			3 UNX + BSA group		
			4 UNX + BSA + MSCs group		
Franchi et al. (2015)	BMMSCs	ARPKD rat model (PCK model)	1 control	2.5 × 10^5^ MSCs intrarenal infusion	↑cortical and parenchymal vasculature density
			2 PCK		→cyst size or number
			3 PCK + MSCs		
Quimby et al. (2016)	ADMSCs	CKD cats	1 MSCs	2×10^6^ MSCs/kg	→Scr, BUN, potassium, phosphorus, GFR by nuclear scintigraphy
			2 Placebo MSCs crossover	IV	No adverse effects
			3 Placebo		
Zhu et al. (2013)	EPC	RAS pigs of renal artery stenosis	1 Intrarenal infusion of vehicle	Vehicle or 10 × 10^6^ EPC or 10×10^6^ MSCs intra-renal infusion	Both EPC and MSCs
	ADMSCs		2 EPC (RAS + EPC)		→renovascular hypertension, PRA, creatinine, and urine protein, Cleaved caspase3
			3 MSCs (RAS + MSC)		↑GFR, post stenotic RBF, transmural cortical microvascular density
			4 normal controls		EPC
Kholia et al. (2020)	BMMSCs- EVs	a mouse model of AAN	1 control group	1 × 10^10^ EV/ml/mouse IV	↓α-SMA, Col1a1, pro-fibrotic genes, blood creatinine and BUN, tubular necrosis, interstitial fibrosis, infiltration of CD45 positive immune cells, fibroblasts, and pericytes
			2 AA group		→weight loss
			3 AA + BMMSCS-EVs group		
Grange et al. (2019)	EVs from bone marrow or liver	NOD SCID gamma mice with STZ-induced T1DM	1 control group	Multiple dose of 1 ×10^10^ (IV)	↓ collagen I, MMP3, TIMP1, FasL, Serpina1a, SNAI1, CCL3, BUN, creatinine, fibrosis, EMT, recruitment of macrophages, T cells
			2 STZ-diabetic mice group		
			3 HLSC EV group		
			4 MSCs EVFIBRO		
			5 EV-treated mice group		
Ramírez-Bajo et al. (2020)	BMMSCs	An aggressive mouse model of chronic CsA nephrotoxicity	1 control group	BMMSCS, EVs and dCM groups were administered as prophylaxis or as treatment of CsA nephrotoxicity	BMMSCs therapy
					↓tubular vacuolization, casts, and cysts rate, BUN
			2 CsA group		↑body weight
	EVs		3 CsA + BMMSCs group		EVs therapy
					↓ the number of cysts
	EVs-depleted CM		4 CsA + EVs group		EVs decreased the BUN levels but without statistical significance
					↑body weight dCM therapy:
			5 CsA + dCM group		↑body weight
					→histological injury
Ishiy et al. (2020)	ADMSCs	2 K-1C rats (model of renal chronic hypoxia)	1 Sham group	The ADMSCs were injected at a density of 2 ×10^5^ cells diluted in 200 μL of PBS. The EV-treated groups received 100 μg of MVs or EVs diluted in 200 μL of PBS	ADMSCs or MV- and EX-treated
			2 Stenotic group		↑SDF1-α(a stem cell homing marker), Col I and TGF-β, IL-10
	ADMSCs-MVs		3 Stenotic + ADMSCs group		↓ HIF1-α, a stabilization of blood pressure
	ADMSCs-EVs		4 Stenotic + MVs group		ADMSCs and MVs
					↓proteinuria
			5 Stenotic + EVs group		ADMSCs
					↑IL-1β
Liu et al. (2018)	UCMSCs-CM	UUO rats	1 sham group	The CM group received UCMSCs-CM (500 μL) *via* left renal artery	↓ malondialdehyde (MDA), reactive oxygen species (ROS), the expression of TGF-β1, α-SMA, TNF-α and Collagen-I, RTEs apoptosis
			2 UUO group		↑activity of glutathione (GSH), proliferation of RTEs, expression of E-cadherin
			3 UUO + CM group		
Wang et al. (2016)	BMMSCs- EVs	UUO mice	1 sham group	EVs released from 1 × 10^6^ MSCs intravenously	↓ fibrosis, collagen, MMP-9, α-SMA, TGF-βR1 its receptor
			2 NTC MSCs group		
			3 miR-let7c MSCs group		
Liu et al. (2021)	UCMSCs -EVs	UUO rats	1 sham operation group	*via* the left renal artery after total ligation of the left ureter	↓apoptosis of NRK-52 E cells, Scr, BUN, oxidative stress, the renal tubular injury and tubulointerstitial fibrosis
			2 sham operation transplanted with UCMSCs -EVs group		
			3 UUO group		
			4 UUO transplanted with UCMSCs -EVs group		

CKD, chronic kidney disease; MSCs, mesenchymal stem cells; BMMSCs, bone marrow mesenchymal stem cells; ADMSCs, adipose-derived mesenchymal stem cells; UCMSCs, umbilical cord blood mesenchymal stem cells; PDMSCs, placenta-derived mesenchymal stem cells; EPC, endothelial progenitor cells; CM, conditioned medium; dCM, exosomes depleted conditioned medium; UCMSCs-Exo, exosomes from umbilical cord mesenchymal stem cells; MVs, microvesicles; SLE, systemic lupus erythematosus; LN, lupus nephritis; DN, diabetic nephropathy; ADPKD, autosomal dominant polycystic kidney disease; ARPKD, autosomal recessive polycystic kidney disease; 5/6 NPX, nephrectomy; UUO, unilateral ureteral obstruction; RAS, renal artery stenosis; AAN, aristolochic acid nephropathy; UNX, uninephrectomy; STZ, streptozotocin; CsA, cyclosporine; 2K-1C, 2 kidneys, 1 clip model; IV, intravenous; α-SMA, α-smooth muscle actin; BMP-7, bone morphogenetic protein 7; BUN, blood urea nitrogen; VEGF, vascular endothelial-derived growth factor; Ccr, creatinine clearance rate; FGFs, fibroblast growth factor; HGF, hepatocyte growth factor; PAI-1, plasminogen activator inhibitor-1; IL-6, interleukin 6; TGF-β1, transforming growth factor-β1; TNF-α, tumor necrosis factor alpha; IL-1β, interleukin-1β; PTC, peritubular capillary; HIF-1α, hypoxia induction factor-1α; IL-10, interleukin 10; PCNA, proliferating cell nuclear antigen; PBS, phosphate-buffered saline; SCr, serum creatinine; UACR, urine albumin creatinine ratio; eGFR, estimated glomerular filtration rate.

### MSCs-EVs and CKD in preclinical study

EVs (extracellular vesicles) are alternative to MSCs. Compared with MSCs, treating CKD with MSCs-EVs may have the advantage of lower immunogenicity, tumorigenicity, and easier management (Abbaszadeh et al., 2020). EVs carry complex cargoes of biological molecules including cytokines, chemokines, growth factors, and nucleic acids. The content of the EVs cargo is related to the state of their cells of origin and can reflect the phenotype of the releasing cell. Many preclinical trials have revealed that MSCs-EVs are effective in treating CKD (Table 1).

In a mouse model, single MSCs-MVs enriched with miR-451a were injected into STZ-induced diabetic nephropathy (DN) animals. The study found that MSCs-MVs enriched with miR-451a decreased the level of α-SMA and increased E-cadherin expression, finally reducing fibrosis (Kholia et al., 2020). BMMSCs-EVs delivered miRNA to mice with STZ induced T1DM and downregulated the expressions of collagen I, metalloproteinase 3 (MMP3), and tissue inhibitor of matrix metalloprotease-1 (TIMP1). Also, the apoptotic gene expressions such as FasL and Serpina1a (α-1-antitrypsinin) were suppressed (Grange et al., 2019). In the mouse model of chronic cyclosporine (CsA) nephrotoxicity, EVs can improve the prognosis of kidney diseases (Ramírez-Bajo et al., 2020).

EVs exert anti-fibrotic activity through the transfer of miRNA targeting predicted pro-fibrotic genes. Multiple studies have demonstrated that MSCs-EVs exert a therapeutic effect by downregulating pro-fibrotic gene expression, inhibiting EMT, reducing tubular atrophy and inflammation, and promoting vascular regeneration. The mechanism of renoprotection of MSCs-EVs was attributed to the downregulation of phosphorylated Smad2, Smad3, and p38 MAPK signaling and upregulation of E-cadherin (Table 1) (Ishiy et al., 2020; Liu et al., 2021).

In a UUO model, the conditional medium (CM) of UCMSCs significantly reduced expressions of pro-fibrogenic factors, such as malondialdehyde, reactive oxygen species, TGF-β1, α-SMA, TNF-α, and Collagen I. Besides, CM increased E-cadherin expression. These data reflect that CM of UCMSCs effectively improves renal interstitial fibrosis induced by UUO by pro-proliferation, anti-apoptosis**,** and anti-fibrosis (Liu et al., 2018). Similarly, a single injection of exosomes enriched with miR-let7 was given to mice with UUO in a study. It found that exosomes with miR-let7 could travel to the kidneys of mice with UUO and downregulate collagen, metalloproteinase-9 (MMP-9), α-SMA, TGF-β type 1 receptor (TGF-βR1) (Wang et al., 2016).

Using MSCs-EVs instead of MSCs would have many advantages in clinical applications, like addressing safety issues such as tumorigenicity, embolisms, and infection transmission through injected cells, as well as making it easier to store them (Vizoso et al., 2017). However, the transition of MSCs-EVs into therapeutically effective drugs offers various hurdles, including determining the optimal method for isolating and characterizing the vesicles in the absence of particular biomarkers. It will be challenging to apply it to future studies due to the lack of an assessment of the complexity of exosome cargo and the possibility of interference by unknown secreted substances. The rules for large-scale manufacturing and quality control are also expected. Moreover, mechanistic studies should also be included in efficacy trials of MSCs-EVs therapy.

## MSCs and CKD in clinical study

### MSCs and DN in clinical study

DM affects millions of people worldwide. Hyperglycemia in DM can affect several major organs. DN is one of the most severe microvascular consequences for both type 1 and type 2 diabetics. Also, DN is still progressing rapidly and is the primary cause of ESRD. Currently, DN is treated by blocking the renin-angiotensin system and controlling glucose, lipids, and blood pressure (Webster et al., 2017). Renal replacement therapy is an effective treatment option for patients with DN who have progressed to ESRD, but it is associated with significant medical and socioeconomic burdens (Tang and Lai, 2012). Therefore, regenerative approaches are urgently needed.

Numerous preclinical trials have indicated that MSCs-based therapy can slow the progression of DN. However, research on MSCs therapy for DN remains limited to preclinical models, and there is very little human data available. Only one clinical trial has been completed so far. In this completed trial, two doses of allogeneic BMMSCs were given to 30 patients with DN. No acute adverse events occurred with the administration of the drug and no patients developed enduring donor-specific anti-HLA antibodies in this study. It is likely that BMMSCs infusion is safe in patients with DN. In addition, the MSCs-treated group showed a more stable or improved eGFR (estimated glomerular filtration rate) at week 12, which may provide a clue to how MSCs act in this disease. In conclusion, this multicenter, randomized, double-blind, dose-escalating, sequential, and placebo-controlled trial suggests that it is feasible and safe to use MSCs in subjects with DN (Table 2) (Packham et al., 2016).

**TABLE 2:** Summary of clinical data of MSCs in CKD.

Author, year	Stem cell type	Patients	Study type	Handling methods	Treatment effect
Packham et al. (2016)	BMMSCs	13 patients at three Australian centers with moderate to severe DN	A multicenter, randomized, double-blind, dose-escalating, sequential, placebo-controlled trial	150×10^6^ or 300×10^6^ MSCs	No acute adverse events
				IV	No patients developed persistent donor specific anti-HLA antibodies
Deng et al. (2017)	UCMSCs	12 among 18 patients were randomized to UCMSCs arm	A randomised double-blind, placebo-controlled trial	2×10^8^ MSCs	No significant difference after the infusion of MSCs
				IV	
Wang et al. (2018)	BMMSCs UCMSCs	81 patients with severe and drug-refractory SLE	A long-term follow-up study	Of the 81 patients, 22 received allogeneic BMMSCs. Four were treated a second time with UCMSCs. Two other patients received two additional doses of UCMSCs and one patient received three additional doses of UCMSCs. Among the 59 patients first treated with UC-MSCs, 7 received a second UCMSCs, 1 received two additional doses of UCMSCs, and 1 received three additional doses of UCMSCs	MSCs transplantation is safe and resulted in long-term clinical remission in SLE patients
Liang et al. (2010)	BMMSCs	15 patients with refractory SLE	A pilot clinical study	13×10^6^/kg	All patients: no serious adverse events, non-renal-related manifestations improved significantly, increase
					2 patients: relapse of proteinuria
				IV	11 patients
					↓SLEDAI score, 24-h proteinuria, anti-dsDNA levels
Yuan et al. (2019)	UCMSCs	21 among 166 SLE patients refractory to conventional therapies were enrolled in UCMSCs therapy	Clinical Trial.gov (identifier: NCT01741857)	2×10^7^/kg	↓Peripheral tolerogenic CD1c+ DCs, serum FLT3L
				IV	↑peripheral blood CD1c+DCs and serum FLT3L
Makhlough et al. (2017)	BMMSCs	6 eligible ADPKD patients	A single-arm phase I clinical trial	2×10^6^/kg	Safe and well tolerated
				IV	→eGFR, BUN, Calcium, Phosphorus, Dipstick proteinuria, Alkaline phosphatase, Total cholesterol, Triglycerides, SCr, Kidney length

BMMSCs, bone marrow mesenchymal stem cells; UCMSCs, umbilical cord blood mesenchymal stem cells; SLE, systemic lupus erythematosus; LN, lupus nephritis; SLEDAI, systemic lupus erythematosus disease activity index; DN, diabetic nephropathy; ADPKD, autosomal dominant polycystic kidney disease; ARPKD, autosomal recessive polycystic kidney disease; IV, intravenous; DCs, dendritic cells; BUN, blood urea nitrogen; eGFR, estimated glomerular filtration rate; SCr, serum creatinine.

### MSCs and LN in clinical study

Systemic lupus erythematosus (SLE) is a chronic inflammatory disease that involves multiorgan damage, including kidneys. LN is a main cause of morbidity and overall mortality in SLE (Almaani et al., 2017). Currently, the efficacy of MSCs in treating LN was controversial.

**FIGURE 1:**
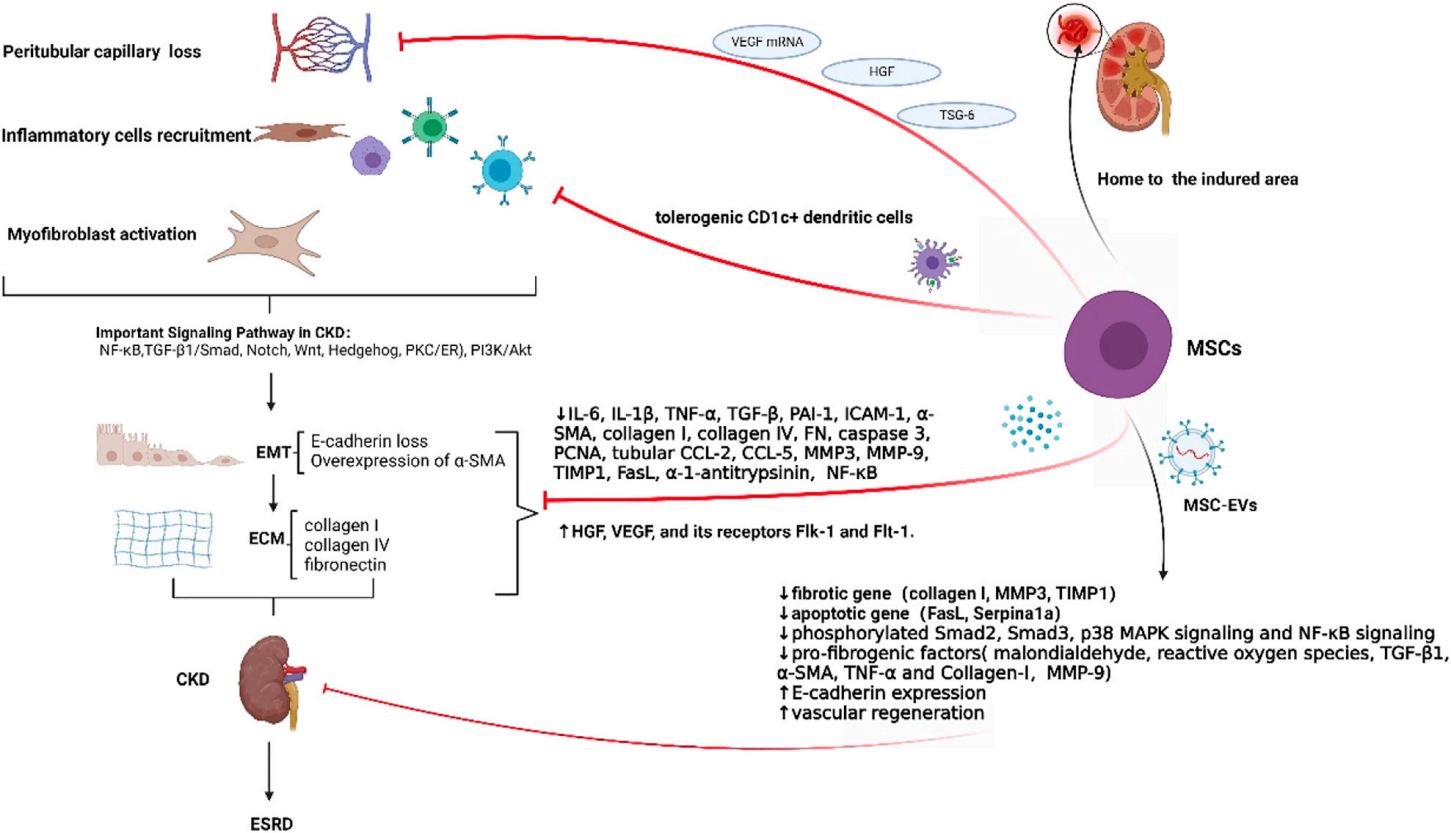
The pathological features of CKD and the functions of MSCs in CKD. Renal fibrosis is a defining feature of CKD. Important pathological changes, in the development of CKD, include the loss of peritubular capillaries, recruitment of inflammatory cells, activation of myofibroblasts, EMT, and ECM deposition. EMT is primarily induced by TGF-β1. Essential markers of EMT include the overexpression of α-SMA and the loss of E-cadherin. Overexpression of fibronectin, collagen I, and collagen IV is a component of ECM deposition. CKD is intimately associated with several signaling networks, including TGF-β, MAPK, Wnt/β-catenin, PI3K/Akt, JAK/STAT, and Notch pathways. In most individuals, multidrug treatment cannot stop the progression toward ESRD. MSCs could reach the injured area to exert renal protective effects. In detail, MSCs could generate VEGF mRNA, which stops the loss of peritubular capillaries. Also, as paracrine factors of MSCs, HGF and TSG-6 could help to reduce peritubular capillaries. Furthermore, MSCs may reduce inflammation by promoting tolerogenic CD1c+ dendritic cells, which can inhibit T cell proliferation and differentiation. Moreover, MSCs could diminish EMT and renal fibrosis, by lowering levels of IL-6, IL-1β, TNF-α, TGF-β, PAI-1, ICAM-1, α-SMA, collagen I, collagen IV, FN, caspase 3, PCNA, tubular CCL-2, CCL-5, MMP3, MMP-9, TIMP1, FasL, α-1-antitrypsinin, phosphorylated Smad2, Smad3, and p38 MAPK signaling. Additionally, MSCs could increase levels of FGFs, HGF, VEGF, VEGF, Flk-1, Flt-1, and E-cadherin, finally slowing down EMT and renal fibrosis. MSC-EVs, carrying complex cargoes of biological molecules (cytokines, chemokines, growth factors, and nucleic acids), could decrease expression of fibrotic genes, apoptotic genes, and pro-fibrogenic factors while increasing E-cadherin expression and vascular regeneration. In conclusion, MSCs can help treat four stages of renal fibrosis: cell damage, activation of fibrogenic signaling, fibrogenic execution, and destruction of fibrogenic tissue.

An RCT study showed that the clinical symptoms and serological markers in the IV type of LN improved with no significant difference after the infusion of MSCs (Deng et al., 2017). However, in a clinical trial involving eighty-one patients with severe and drug refractory SLE, MSCs were safe and resulted in long-term clinical remission in SLE patients. Moreover, at follow-up after 1, 2, 3, 4, and 5 years, 24-h proteinuria was significantly reduced after MSCs treatment (Wang et al., 2018). In another clinical study, a single injection of allogeneic BMMSCs was given to patients with refractory SLE. Although two patients had a relapse of proteinuria, the remaining 11 patients showed a significant decrease in proteinuria and also an improvement in serological markers. Increased levels of circulating CD4^+^ Foxp3+ Tregs induced by BMMSCs may contribute to these positive findings (Liang et al., 2010). However, the exact mechanism was unclear. Allogeneic UCMSCs might suppress inflammation in SLE patients by up-regulating tolerogenic CD1c+ dendritic cells, suppressing T cells proliferation and differentiation (Yuan et al., 2019). Despite the *in vivo* evidence for the therapeutic efficacy of MSCs in LN treatment, the underlying mechanisms remain unclear. Therefore, future advances can explore specific mechanisms of MSCs behaviors in LN patients (Figure 1 and Table 2).

### MSCs and ADPKD in clinical study

ADPKD is the most common genetic cause of CKD with chronic nonproteinuric nephropathies (Patel et al., 2009). Current management for ADPKD includes blood pressure control, dietary sodium restriction, and increasing fluid intake. Potential treatments for ADPKD include tolvaptan, octreotide, cystic fibrosis transmembrane conductance regulator protein (CFTR) inhibitors, pioglitazone, etanercept, and triptolide (Patel et al., 2009). A safety assessment of autologous BMMSCs has been conducted in a phase 1 clinical trial including six patients with ADPKD. This report indicated that no cell-related adverse events were observed following a single-cell infusion of MSCs during the 12-months follow-up. This trial demonstrated that the mean serum creatinine (SCr) level of the patients gradually increased from 2 ± 0.3 mg/dl to 2.5 ± 0.4 mg/dl before MSCs infusion and remained stable at 2.5 ± 0.6 mg/dl after 12 months of BMMSCs treatment. However, BMMSCs infusion did not significantly affect eGFR or kidney length, an index of kidney growth. These non-significant findings may be associated with the small number of patients, the short follow-up period, the single cell infusion, or the unexplored possible dysfunction of autosomal MSCs derived from patients with ADPKD (Makhlough et al., 2017). There is still relatively little research on MSCs therapy for ADPKD. More research is expected to validate the therapeutic effects of MSCs (Table 2).

### Challenges and future perspectives for MSCs therapy

MSCs might be an attractive treatment for CKD. However, low cell survival after transplantation restricts the efficiency of cell therapy. We cover the major barriers to successful cell engraftment as well as the solutions that have been investigated to increase the therapeutic benefits of cell therapy.

The number of given cells that reach the target area, their vitality, and their potential to promote tissue regeneration will ultimately decide the cell therapy efficacy. Several variables influence long-term transplanted cell survival, including mechanical stress during implantation, extracellular matrix loss during delivery, nutrition and oxygen deprivation at the recipient site, increasing age, and pathological circumstances.

Cell membranes are easily ruptured by the mechanical stresses during injection, in particular the stretching and shearing forces created by the extensional and linear flow into the syringe needle or catheter (Aguado et al., 2012). Cell injection techniques usually result in a loss of living cells, with viabilities ranging from 1 to 32% post-transplantation (Zhang et al., 2001). Therefore, improvement of the delivery techniques may greatly enhance the vitality and function of the injected cells. Hydrogels may aid in increasing the viscosity of the cell suspension, hence lowering the mechanical forces during injection (Foster et al., 2017).

ECM facilitates cell adherence and engraftment (He et al., 2015). However, therapeutic applications of MSCs are based on single cell suspension, which results in the loss of cell-matrix connections and the downregulation of adhesion signals, leading to cell death (He et al., 2015).

Oxygen and nutrition deprivation caused by delayed revascularization at the implantation site is a key cause of early cell death (Rouwkema et al., 2010). Several preconditioning procedures have been devised to increase the resistance of transplanted cells to death triggers after transplantation. One technique is to stimulate a wide prosurvival response in cells by subjecting them to a physical or environmental shock, such as high temperature, nutritional restriction, hypoxia, or anoxia (Fulda et al., 2010). Sub-lethal settings enable cells to progressively adapt to environmental changes, generating an anti-stress response and activating pathways that facilitate survival (Fulda et al., 2010).

The proliferation and differentiation abilities of MSCs significantly decline with increasing age (Li et al., 2014). The poor health of the patient may also limit the function of MSCs derived from CKD patients for the use of autologous MSCs. Furthermore, diabetes also creates an unfavorable microenvironment for MSCs, making it harder to survive, migrate to infected tissue, and perform their functions. *In vivo*, MSCs show reduced proliferation and viability with a reduced level of proteoglycans and glycosaminoglycans in the surrounding tissue (Bi et al., 2005). The production of advanced glycosylated end products also triggers apoptosis and the generation of reactive oxygen species, inhibiting MSCs proliferation. Oxidative stress can also affect the paracrine effects of MSCs under hypoxic conditions in diabetics. Superoxide levels in hypoxic MSCs rise a lot, which makes them less likely to make angiogenic growth factors like hypoxia-induced factor-1α, VEGF-A, and platelet-derived growth factor B (PDGF-B) (Kume et al., 2005; Ishizuka et al., 2011). MSCs are also impaired in their migration ability in high glucose conditions (Secchiero et al., 2008). As a result, novel approaches are required to improve the therapeutic efficacy of MSCs in pathophysiological conditions. Several studies have shown that preconditioning of MSCs extends their lives and improves their function. Pretreatment can be achieved by using specific compounds such as MT and fucoidan. Pretreatment with MT ameliorated oxidative stress and senescence in a model of ischemic disease associated with CKD (Han et al., 2019). Additionally, fucoidan-treated MSCs can enhance cell proliferation, angiogenesis, and regeneration in a model of hind limb ischemia associated with CKD in mice (Han et al., 2015).

**FIGURE 2:**
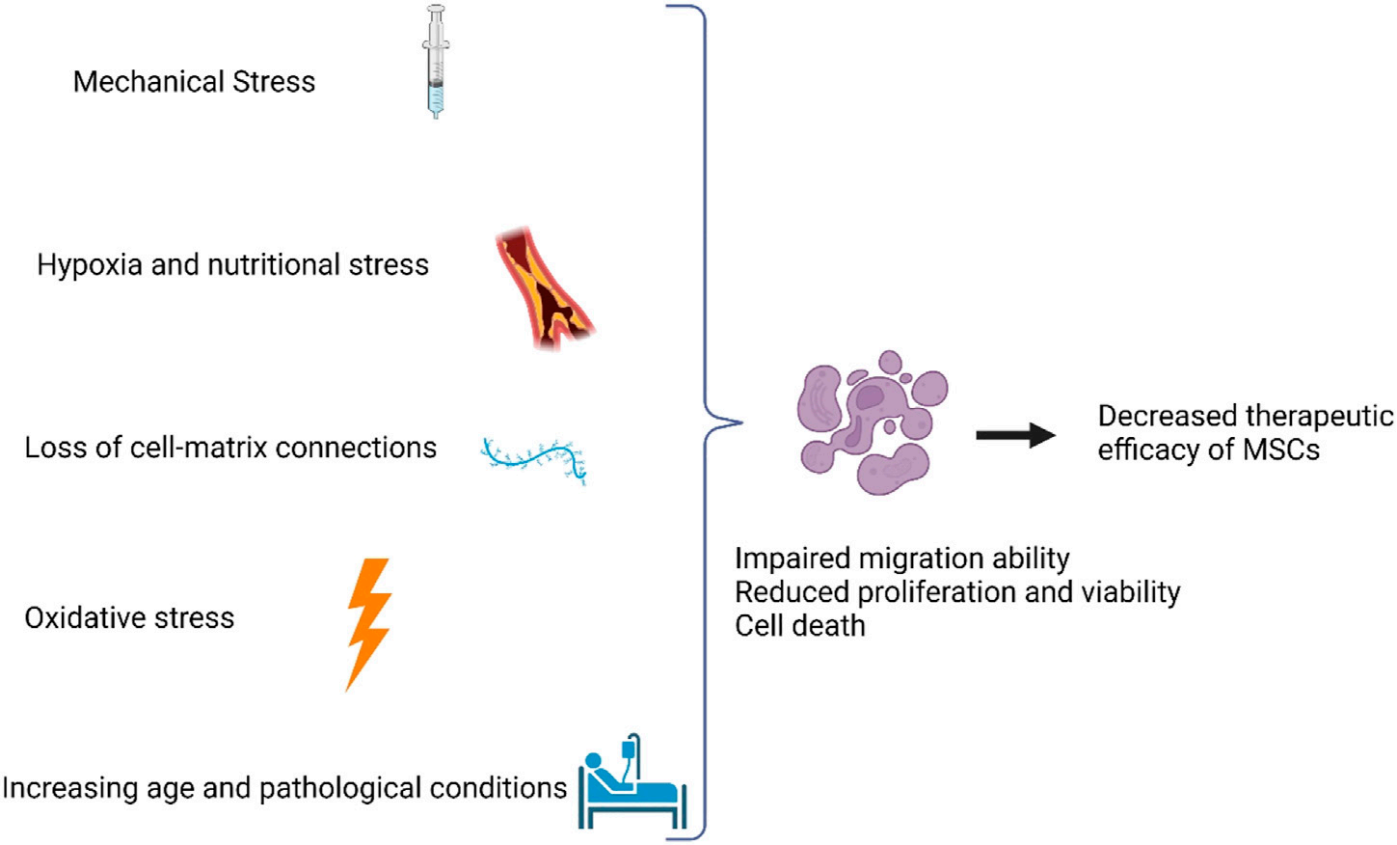
Factors limiting therapeutic efficiency of MSCs. Factors that affect the therapeutic benefit include mechanical stress, hypoxia and nutritional stress, loss of cell-matrix connections, oxidative stress, increasing age, and pathological conditions.

In conclusion, reduced cell survival after transplantation limits the efficacy of cell treatment, which is caused by a combination of mechanical, cell, and human factors. Many techniques have been developed to promote the survival of MSCs after transplantation in preclinical studies, such as hydrogel cicrocarriers, biomaterials, preconditioning, and so on. However, before these strategies can be successfully used in the clinic, more studies are needed to find out if and how they affect the biological activity of transplanted cells *in vivo* (Figure 2).

## Discussion

Various studies have proposed that MSCs can reduce the development and progression of CKD by modulating immunity, inhibiting inflammatory factors, and improving renal function (Ruan et al., 2014; Ishiy et al., 2020; Xiang et al., 2020). MSCs could reduce tubular EMT and renal fibrosis by decreasing levels of IL-6, IL-1β, TNF-α, TGF-β, PAI-1, ICAM-1, α-SMA, collagen I, collagen IV, FN, caspase 3, PCNA, tubular CCL-2, CCL-5, MMP3, MMP-9, TIMP1, FasL, α-1-antitrypsinin, phosphorylated Smad2, Smad3, NF-κB expression, and p38 MAPK signaling. However, levels of FGFs, HGF, VEGF, HGF, VEGF, Flk-1, Flt-1, and E-cadherin were increased in this process.

Using MSCs for CKD therapy has been demonstrated to be feasible and safe based on many experimental evidence and clinical trials. Concerns about an increase in the risk of opportunistic infections have been alleviated by reassuring safety data from a large kidney transplant program (Reinders et al., 2013). The reassuring safety profile of MSCs in patients with kidney disease is consistent with other published information (Casiraghi et al., 2013), but long-term monitoring of MSC-treated patients with nephropathy is still essential, especially for the potential risk of developing anti-HLA antibodies and cancer.

Data on efficacy are promising, however, the results have not been replicated in the clinic (Bao et al., 2018). In STZ-induced DN rats, MSCs were able to migrate to the kidneys and repair renal dysfunction effectively, such as lowering proteinuria, Scr, and urea nitrogen levels and increasing creatinine clearance rate (Ccr) (Xiang et al., 2020). However, in 30 DN patients, no functional parameters were improved except a more stable or improved eGFR at week 12 after using MSCs (Packham et al., 2016).

Differences in animal models and human physiology preclude the direct translation of promising results from animal experiments into clinical applications. In animal models, kidney diseases are typically induced artificially. Generally, induced injury is acute, unphysiological, and does not illustrate the complex pathophysiology of the human kidney (Pesenti et al., 1990; Hewitson et al., 2015). Thus, it is difficult to predict precisely how a disease will respond to treatment using an animal model. Because other factors like age, gender, and comorbidities are not considered in animal experiments, CKD animal experiments are not sufficiently reflective of the conditions of the disease. Additionally, CKD patients may suffer from comorbidities affecting multiple organs and functions, further triggering the pathological processes underpinning CKD (Togoe et al., 2014). These complications have not been considered in animal models.

The potential use of EVs as an alternative to live MSCs should also be explored. Considering the complexity of exosome cargo and the possibility of interference by unknown secreted substances, future studies will be challenging to apply. Nevertheless, quality control and large-scale manufacturing rules remain unclear. The hurdles above will require effort and attention, but they are not unimprovable.

## Conclusion

There has been extensive preclinical and clinical data showing that cell-based therapies using MSCs can modulate immunity, inhibit inflammatory factors, and improve renal function in CKD, suggesting the possibility of MSCs as a new, effective therapeutic tool for CKD. However, more well-designed studies in preclinical and clinical studies should be performed in the future to confirm it.

## Glossary

ADMSCs: adipose-derived mesenchymal stem cells
ARPKD: autosomal recessive polycystic kidney disease
ADPKD: autosomal dominant polycystic kidney disease
BMMSCs: bone marrow mesenchymal stem cells
BMP-7: bone morphogenetic protein seven
CKD: chronic kidney disease
Ccr: creatinine clearance rate
CM: conditional medium
CsA: cyclosporine
CMV: cytomegalovirus
CFTR: cystic fibrosis transmembrane conductance regulator protein
DM: diabetes mellitus
DMSCs: dental mesenchymal stem cells
DN: diabetic nephropathy
ESRD: end-stage kidney disease
EMT: epithelial-mesenchymal transition
ECM: extracellular matrix
EVs: extracellular vesicles
eGFR: estimated glomerular filtration rate
FGF: fibroblast growth factor
FGFs: fibroblast growth factor
FN: fibronectin
HGF: hepatocyte growth factor
ISCT: International Society of Cell Therapy
IL-6: interleukin six
IL-1β: nterleukin-1β
LN: lupus nephritis
MAPK: mitogen-activated protein kinase
MSCs: mesenchymal stem cells
MT: melatonin
MMP3: metalloproteinase three
MPC: mesenchymal precursor cells
MMF: mycophenolate mofetil
PCNA: proliferating cell nuclear antigen
PDMSCs: placenta-derived mesenchymal stem cells
PKD: polycystic kidney disease
PDGF-B: platelet-derived growth factor B
SGLT2: sodium-glucose cotransporter two
SCr: serum creatinine
STZ: streptozotocin
SLE: systemic lupus erythematosus
TGF-β1: transforming growth factor-β1
TSG-6: tumor necrosis factor stimulated gene-6
TNF-α: tumor necrosis factor alpha
TIMP1: tissue inhibitor of matrix metalloprotease-1
UCMSCs: umbilical cord mesenchymal stem cells
UUO: unilateral ureteral obstruction
VEGF: vascular endothelial growth factor
WJMSCs: wharton’s jelly-derived mesenchymal stem cells
α-SMA: α-smooth muscle actin
5/6 NPX: nephrectomy
